# Novel Algorithm for Identifying and Fusing Conflicting Data in Wireless Sensor Networks

**DOI:** 10.3390/s140609562

**Published:** 2014-05-30

**Authors:** Zhenjiang Zhang, Tonghuan Liu, Dong Chen, Wenyu Zhang

**Affiliations:** School of Electronic and Information Engineering, Key Laboratory of Communication and Information Systems, Beijing Municipal Commission of Education, Beijing Jiaotong University, Beijing 100044, China; E-Mails: 11120129@bjtu.edu.cn (T.L.); 12120202@bjtu.edu.cn (D.C.); 13120179@bjtu.edu.cn (W.Z.)

**Keywords:** wireless sensor network data fusion, keyword, DS theory, evidential conflict

## Abstract

There is continuously increasing interest in research on multi-sensor data fusion technology. Because Dempster’s rule of combination can be problematic when dealing with conflicting data, there are numerous issues that make data fusion a challenging task, including the exponential explosion, Zadeh Paradox, and one-vote veto. These issues lead to a great difference between the fusion results and real results. This paper applies the idea of analyzing distance-based evidence conflicts, introduces the concept of vector space, and proposes a new cosine theorem-based method of identifying and expressing conflicting data. In addition, this paper proposes a new data fusion algorithm based on the degree of mutual support between beliefs, which is based on the Jousselme distance-based combination rule proposed by Deng *et al.* Simulation results demonstrate that the presented algorithm achieves great improvements in both the accuracy of identifying conflicting data and that of fusing conflicting data.

## 1. Introduction

The Dempster–Shafer (DS) theory, which is also known as the evidential theory, can be considered to be a generalization of the Bayesian theory. The DS theory uses a collection to construct the general framework of an uncertainty reasoning model rather than using the specific probability to describe the uncertainty. By establishing the corresponding relation between hypotheses and collections, the uncertainty of hypotheses can be transformed into the corresponding collections. Under the circumstances that *a priori* probabilities are hard to achieve, the evidential theory can distinguish between the uncertain and the unknown, and thus it has more flexibility.

In the field of multi-sensor data fusion, the DS theory is a popular method to express and fuse the uncertainty information, and is especially suitable for decision level fusion. However, the appearance of conflicting evidence results in a series of problems when conducting fusion using the DS theory. Zadeh discovered that simply using Dempster’s rule of combination to deal with highly conflicting data will produce some counter-intuitive results. This is known as Zadeh’s Paradox [1]. In addition, there is another problem called the one-vote veto; namely, when the description of one piece of evidence is completely inconsistent with other pieces of evidence for the same hypothesis (in other words, the belief in the hypothesis is 0), whatever the belief in the other pieces of evidence for the hypothesis, the belief in the fusion results computed by Dempster’s rule of combination will always be 0. The one-vote veto issue brings about the following problem: because of the conclusion of one piece of evidence, the belief in other pieces of evidence is invalid for the final fusion result. Because this problem is caused by conflicting evidence, there is a real need to identify and express highly conflicting data before using the DS theory.

This paper applies a distance-based idea to deal with evidence conflict and introduces the concept of vector space. On this basis, we propose a cosine theorem-based method that can effectively identify and explicitly express the conflicting data. On the basis of the above, we propose a new multi-sensor data fusion algorithm based on the degree of mutual support between beliefs, which can solve the data conflict.

The rest of this paper is organized as follows: in Section 2, we introduce the background and related work. The cosine theorem-based method for identifying and expressing conflicting evidence is described in Section 3. Section 4 provides the data fusion algorithm based on the degree of mutual support between beliefs on the basis of Section 3, and simulation verification is performed in Section 5. In Section 6, we present the concluding remarks for this paper, as well as the outlook of our future work.

## 2. Related Work

The DS theory uses Θ to denote a set of mutually exclusive and independence hypotheses about some problem domain. Thus, Θ is composed of all the possible answers to a question and is called the sample space or frame of discernment, which is indicated as Θ = {Θ_1_, Θ_2_,…, Θ_n_}. We can define *P*(Θ) as the set of all possible combinations of all the elements, which is denoted as P(Θ) = {Ø, {Θ_1_},{Θ_2_},…,{Θ_n_}, { Θ_1_∪Θ_2_}, { Θ_1_∪Θ_3_},…, Θ}. We define a function *m* from *P*(Θ) to [0, 1] such that:

(1) $$\left[m\left(\emptyset \right)=0,\sum_{A\in P\left(\Theta \right)}m\left(A\right)=1\right]$$

Then, function *m* is called the basic probability assignment (BPA), the quantity *m*(*A*) represents the measure of belief that is committed exactly to hypothesis A, and A is called the focal element If *m*(*A*) > 0.

Note that *m*_1_, *m*_2_ are two basic probability assignments of discernment frame Θ, for ∀ *A*⊆Θ, and the combination of *m*_1_, *m*_2_ is given by the following formula, which is called Dempster’s rule of combination:

(2) $$\left[m\left(A\right)=m_{1}⊕m_{2}\left(A\right)=\frac{1}{1-K}\underset{B\cap C=A}{\text{∑}}m_{1}\left(B\right)\cdot m_{2}\left(C\right)\right]$$

where 1−*K* is called the normalization constant, and such that:

(3) $$\left[K=\underset{B\cap C=\emptyset }{\text{∑}}m_{1}\left(B\right)\cdot m_{2}\left(C\right)\right]$$

However, the conflict data brings a challenge for the application of the DS theory. Many researchers have been researched the conflict data of the evidence theory, there has been no clear definition. Here, we introduce a simple and intuitive definition of the conflict data.

### Definition 1

(The notion of conflict): in DS evidence theory, the conflict between two reliabilities can be qualitatively expressed as a source of evidence strongly supports a hypothesis, but another source of evidence strongly supports an alternative hypothesis, and the two assumptions are incompatible.

In fact, the degree of conflict between the evidences can also be said the degree of mutual support. If the degree of conflict is small, it means conclusions of the evidence can support each other; otherwise, it means they cannot support each other. There have been some improvements and schemes resolving conflict data problems when using Dempster’s rule of combination. The existing research can be approximately classified into two aspects: one is the improvement of the analysis, judgment, and expression of conflict evidences, and the other is the amelioration of the fusion algorithm for conflicting data.

### 2.1. Research on Analysis, Judgment, and Expression of Conflicting Evidence

As the fusion of conflicting data can be problematic when using Dempster’s rule of combination, it is very important to reasonably and effectively judge the degree of data conflict. There have been various studies on this topic.

#### 2.1.1. Conflicting Coefficient-Based Expression Methods

The earlier analysis of conflicting data was based on the quantity *K* computed by Dempster’s rule of combination. It can be observed from the formula that *K* is calculated by the sum of the products of every two BPAs of completely conflicting propositions B and C. This is called the conflicting coefficient, the value of which can partly reflect the proportion of uncertain information caused by conflicting evidence. However, it has been proven from practical experience that there is no direct relationship between the value of *K* and the conflicting relation of two pieces of evidence. Thus, it is unreasonable to measure the degree of conflict between pieces of evidence using quantity *K*.

#### 2.1.2. Distance-Based Conflict Expression Methods

Jousselme *et al.* introduced the notion of vector space for evidential theory to propose a conflict expression method based on the distance between the pieces of evidence [2]. The authors proposed to regard every basic probability assignment as a multi-dimensional vector. Thus, the degree of conflict between different pieces of evidence can be measured by the distance between the corresponding vectors, with a greater the distance indicating a higher degree of conflict. In contrast, a shorter distance indicates a lower degree of conflict and a better degree of mutual support between pieces of evidence.

##### Definition 2

(Jousselme distance): let Θ be the frame of discernment composed of n mutually exclusive and exhaustive hypotheses. *m*_1_, *m*_2_ are two basic probability assignments of discernment frame Θ; and the distance between two pieces of evidence for *m*_1_ and *m*_2_ is found as follows:

(4) $$\left[d_{\text{BPA}}\left(m_{1},m_{2}\right)=\sqrt{½{\left(\vec{m_{1}}-\vec{m_{2}}\right)}^{T}\underset{=}{D}\left(\vec{m_{1}}-\vec{m_{2}}\right)}\right]$$

Here, $\vec{m_{1}}$ and $\vec{m_{2}}$ are vector representations of *m*_1_ and *m*_2_, and the dimensionality is 2*^N^*. Meanwhile, $\underset{=}{D}$ is a 2*^N^* × 2*^N^* matrix, the elements of which are calculated as follows:

(5) $$\left[\begin{array}{cc} D\left(A,B\right)=\frac{\left|A\cap B\right|}{\left|A\cup B\right|}, & A,B\in P\left(\Theta \right) \end{array}\right]$$

The introduction of matrix $\underset{=}{D}$ has the advantage of taking the similarity between the basic probability assignments into consideration. Hence, it avoids the disadvantage of Euclidean distance. However, the complexity of the algorithm will be very high because of the great size of matrix $\underset{=}{D}$.

#### 2.1.3. Pignistic Probability-Based Conflict Expression Methods

Some researchers have started from the results of the basic probability assignments and proposed Pignistic probability-based conflict expression methods.

##### Definition 3

(Pignistic probability function) [3]: Suppose *m* is the basic probability assignment of sample space Θ, and *A* is a subset of Θ. Then, the Pignistic probability assignment function *BetP_m_* : *θ* → [0, 1] is defined as follows:

(6) $$\left[{\text{BetP}}_{m}\left(w\right)=\underset{A\subseteq \Theta ,w\in A}{\text{∑}}\frac{1}{\left|A\right|}\frac{m\left(A\right)}{1-m\left(\emptyset \right)},m\left(\emptyset \right)\neq 1\right]$$

Here, |*A*| indicates the number of elements in *A*. The Pignistic probability of the subset *A* is:

(7) $$\left[{\text{BetP}}_{m}\left(A\right)={\text{∑}}_{w\in A}{\text{BetP}}_{m}\left(w\right)\right]$$

The transformation from basic probability assignment *m* to the Pignistic probability is called the Pignistic probability transformation. Pignistic probability can effectively reflect the belief assignment of a given hypothesis, because it takes into consideration the influence of the inclusion relation between hypotheses on the probability.

#### 2.1.4. Gambling Credibility Distance-Based Conflict Expression Methods

On the basis of Pignistic probability-based conflict expression methods, Liu proposed using the concept of using the gambling credibility distance to express the degree of conflict [4]. The gambling credibility distance lives up to the original definition of the judgment of an evidence conflict by judging the degree of evidence conflict through determining the maximum difference in the degrees of support given by different pieces of evidence to the hypothesis. If the difference is relatively great, that suggests that the difference between pieces of evidence is larger, which indicates the existence of conflict. In contrast, if the maximum difference is very small, this means the conflict between the pieces of evidence is small. The computation complexity of this method is significantly less than the Jousselme distance-based method, but it has the disadvantage of not being sensitive enough to the degree of conflict.

##### Example 1

The following shows the basic probability assignments for pieces of evidence *E*1, *E*2 and *E*3 under discernment frame Θ:

$$\begin{array}{c} \begin{array}{cccc} E1: & m_{1}\left(A\right)=0.6, & m_{1}\left(B\right)=0.2, & m_{1}\left(C\right)=0.1\\ E2: & m_{2}\left(A\right)=0.1, & m_{2}\left(B\right)=0.4, & m_{2}\left(C\right)=0.5\\ E3: & m_{3}\left(A\right)=0.1, & m_{3}\left(B\right)=0.6, & m_{3}\left(C\right)=0.3 \end{array}\\ \begin{array}{cccc} E1: & m_{1}\left(A\right)=0.6, & m_{1}\left(B\right)=0.3, & m_{1}\left(C\right)=0.1\\ E2: & m_{2}\left(A\right)=0.1, & m_{2}\left(B\right)=0.4, & m_{2}\left(C\right)=0.5\\ E3: & m_{3}\left(A\right)=0.1, & m_{3}\left(B\right)=0.6, & m_{3}\left(C\right)=0.3 \end{array} \end{array}$$

According to the concept of the gambling credibility distance, *A*, *B* and *C* of this example are singleton sets and have no intersection. Thus, the Pignistic probability is the same as the belief assignment result. We can see that the degrees of conflict for the two methods are the same. However, even though the maximum difference in Pignistic probability between *E*2 and *E*1 equals that between *E*3 and *E*1, it is observed that on the condition that the belief assignment result for *A* of *E*3 remains the same, the belief assignment result for *B* increases. Thus, *E*3 is approximately closer to *E*1, and the degree of conflict is smaller than that between *E*2 and *E*1.

#### 2.1.5. Axiomatic Definition of Conflict between Belief Functions

Destercke and Burger [5] proposed to measure the conflict between two bodies of evidence from a different perspective. He started by examining consistency and conflict on sets and extract from this settings basic properties that measures of consistency and conflict should have. They then extend this basic scheme to belief functions in different ways. Two main ideas motivate this study and its results. The first is the idea that the properties of conflict measures between belief functions should be based on properties that appear natural when measuring conflict between sets. The second is the idea that a measure of conflict between sources should not depend *a priori* on a specific dependence assumption between the sources.

Conflict k for sets should be such that:

(8) $$\text{k}\left(A,B\right)=1-\emptyset \left(A\cap B\right)=\{\begin{array}{cc} 1, & \text{if A}\cap \text{B}=\emptyset \\ 0, & \text{if A}\cap \text{B}\neq \emptyset \end{array}$$

In particular, they do not make any *a priori* assumption about sources dependence and only consider such assumptions as possible additional information.

#### 2.1.6. A New Combination Rule to Keep the Initial Meaning of the Conflict

Lefèvre and Elouedi [6] proposed a combination rule (CWAC) to preserve the main role of a conflict as an alarm signal indicating that there is a kind of disagreement between sources. The combination rule provided an adaptive weighting between Dempster’s rule and conjunctive rule, which enables keep the initial meaning of the conflict obtained during the combination and thus to restore its initial role of alarm. Thus, it permits the conflict to take back its initial sense by only mentioning that there is a problem.

#### 2.1.7. Using Discounting Rate to Reduce the Weight of the Evidence

Klein and Colot [7] used the evidential conflict analysis to mine singular sources. They regarded the degree of conflict as a function of parameters called discounting rates, thus to introduced a criterion to weights the contribution of each basic belief assignment (bba). The bba will be assigned a large discounting rate when a source is unreliable so that its weight in the combination process is reduced. The proposed criterion was more robust to parameters such as the proportion of conflicting bbas and the number of bbas with a better or equivalent efficiency.

### 2.2. Research on Fusion Algorithm for Conflicting Data

Nowadays, researchers at home and abroad mainly focus on two aspects of fusion algorithms: improving Dempster’s combination rule and modifying the evidence source model.

#### 2.2.1. Improvement in Dempster’s Combination Rule

When the pieces of evidence are highly conflicting, Dempster’s combination rule normalizes the conflicting data directly without a reasonable belief assignment, which leads to unreasonable results after data fusion. In view of this problem, some researchers have proposed new combination rules.

Yager considered the discernment frame to be exhaustive under a closed world assumption. Therefore, the conflicting data should be regarded as unknown and uncertain information and contained in *m*(Θ). This removes the regularization process of the original combination rule [8]. In contrast, Smets held the view that the conflict is also a kind of information, the cause of which is an incomplete discernment frame for an unknown environment. Thus, Smets assigned the conflicting data to the focal element for a combined empty set, and then proposed a transferable belief model [9]. Doubois and Prade suggested assigning the conflicting data to the union set of conflicting focal elements [10]. Lefevre *et al.* focused on the assignments of conflicting pieces of evidence, and proposed a generic form for conflicting evidence assignment after synthesizing several methods for conflict resolution [11]. Later studies were primarily based on the above-mentioned ideas.

#### 2.2.2. Improvement of Evidence Source Model

Other researchers maintained that the unreasonable fusion results come from direct fusion without preprocessing the conflicting data.

Some representative methods are as follows: Shafer introduced a discount factor to modify the mass function when some uncertain information is given [12]. Murphy put forward the average evidence combination rule, after analyzing the existing methods [13]. However, the average combination rule simply considers the average for the pieces of evidence without taking the correlation between them into consideration. Thus, it is sensitive to the influence of data error. On the basis of Murphy’s method, many improved methods have been proposed. Deng *et al.* [14] introduced the Jousselme distance, which was introduced in Section 2.1, into Murphy’s method to obtain the weighted average of multi-source evidence, the weight of which depends on the mutual degree of support between pieces of evidence. Han *et al.* [15] took the uncertainty of evidence into consideration based on the foundation of Deng’s work, but it is unreasonable to measure the credibility of the evidence by the belief assignment result. For example, *m*_1_ (*A*) = 0.5 *m*_1_ (*B*) = 0.5, *m*_2_ (*A*) = 0 *m*_2_ (*B*) = 1. It is unreasonable to consider that *m*_2_ is more credible, for the uncertainty of the reliability allocation results of *m*_2_ is smaller than *m*_1_. Liu *et al.* [16] suggested using the gambling credibility distance and conflicting coefficient introduced above to modify the mass function.

In general, there are deficiencies in all of the existing methods for improving the analysis, judgment, and expression of conflicting data, as well as the fusion algorithm for such data. In the following section, we will introduce our solution to deal with conflicting data.

### 2.3. Open World Assumption and Closed World Assumption

In brief, Closed World Assumption means that all situations are known in the current environment and the Open World Assumption means that there are unknown situation in the current environment. In evidence theory, there is a special condition: *m*(Ø) = 0. And this assumption belongs to the Closed World Assumption, for all reliabilities are allocated in the known identification framework. But in the Transferable Belief Model (TBM), which is proposed by Smets and Kennes [17], the value of *m*(Ø) can be larger than zero. This means the reliabilities are distributed beyond the identification framework. Thus, the evidence theory application has been generalized to the Open World Assumption. In this article, we will discuss basic reliability allocation policy in Open and Closed World Assumption respectively.

## 3. Cosine Theorem-Based Method for Identifying and Expressing Conflicting Data

This paper references Jousselme’s idea of using distance to analyze conflicting pieces of evidence. We introduce the concept of vector space and combine it with the angle concept from the cosine theorem to analyze and identify the conflicting evidence.

Considering the computing complexity of a data conflict expression method, this paper suggests using an n-dimensional vector space, where n is the number of elements from discernment frame Θ, which is more efficient than the 2^n^-dimensional vector space proposed by Jousselme. In addition, for the open world assumption, an n + 1-dimensional vector space for analyzing the conflicting evidence is needed because there may be a belief assigned to the empty set.

For a discernment frame Θ that contains n elements, there are 2^n^ possible hypotheses in the problem domain. Therefore, we should perform the transition from a 2^n^-dimensional belief assignment vector to an n-dimensional vector, where the converted n-dimensional vector is obtained from the Pignistic probability function introduced in Definition 3.

Assume that Θ = {Θ_1_, Θ_2_,…, Θ_n_}, namely n mutually exclusive and exhaustive elements; *m* is the basic probability assignment of 2^n^ hypotheses; and the n-dimensional Pignistic probability vector of *m* is calculated by Formula (7):

(9) $$\left[{\text{PignisticVector}}_{m}=\left(\begin{array}{c} \underset{A\subseteq \Theta ,A\cap \Theta_{1}\neq \emptyset }{\text{∑}}\frac{1}{\left|A\right|}\frac{m\left(A\right)}{1-m\left(\emptyset \right)}\\ \underset{A\subseteq \Theta ,A\cap \Theta_{2}\neq \emptyset }{\text{∑}}\frac{1}{\left|A\right|}\frac{m\left(A\right)}{1-m\left(\emptyset \right)}\\ \vdots \\ \underset{A\subseteq \Theta ,A\cap \Theta_{i}\neq \emptyset }{\text{∑}}\frac{1}{\left|A\right|}\frac{m\left(A\right)}{1-m\left(\emptyset \right)}\\ \vdots \\ \underset{A\subseteq \Theta ,A\cap \Theta_{n}\neq \emptyset }{\text{∑}}\frac{1}{\left|A\right|}\frac{m\left(A\right)}{1-m\left(\emptyset \right)} \end{array}\right)\right]$$

Equation (9) accomplishes the transition from the 2*^n^*-dimensional belief assignment vector to the *n*-dimensional vector. Under the open world assumption, to distinguish from the conflicting coefficient *K* of Dempster’s combination rule, we denote the *n* + 1 dimensional of PignisticVectorm, which expresses the Pignistic probability of hypotheses out of the discernment frame by *m*(*φ*).

According to the definition of evidence theory, the basic probability assignment m will be greater than or equal to 0 and less than or equal to 1. Therefore, the Pignistic probability of n elements from Θ is still equal to 0 and less than or equal to 1 when the transition is completed to the *n*-dimensional vector of PignisticVectorm.

Above all, the belief assignment result for every piece of evidence will be mapped to an *n*-dimensional vector starting from the origin and ending with the vector calculated by Equation (7).

**Figure 1.**
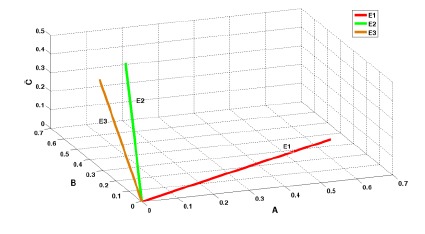
Vector space graph of belief assignment function in Example 1.

According to Example 1, in a case where the PignisticVector is the same as the original basic probability assignment, the corresponding vector space is shown in Figure 1. We have completed the transition to the n-dimensional vector of the belief assignment result. According to the definition of the basic probability assignment, the sum of the assignment results equals 1. Therefore, the sum of n Pignistic probabilities of PignisticVectors from the same evidence will also be 1, which ensures that different PignisticVectors map to different space vectors (there is a certain angle between vectors). This fits the intuitive understanding that only identical assignment results infer that there is no conflict. Therefore, using the concept of angle to judge the degree of conflict between pieces of evidence is reasonable in terms of intuitive sense. The following shows a more detailed analysis.

As the space vector coordinates are known, it is easy to get the angle between the vectors using the cosine theorem. For discernment frame Θ = {Θ_1_, Θ_2_,…,Θ_n_}, *m*_1_, *m*_2_ are two basic probability assignments of Θ. We can get *PignisticVe*c*tor m*_1_ and *PignisticVe*c*tor m*_2_ using Formula (9). Then, the cosine of the angle between *PignisticVe*c*tor m*_1_ and *PignisticVe*c*tor m*_2_ can be calculated by:

(10) $$\left[\cos\left(m_{1},m_{2}\right)=\frac{<\text{PignisticVector}\;m_{1},\text{PignisticVector}\;m_{2}>}{\left|\text{PignisticVector}\;m_{1}\right|\times \left|\text{PignisticVector}\;m_{2}\right|}\right]$$

Here, <*PignisticVector m*_1_, *PignisticVector m*_2_> indicates the dot product of *PignisticVector m*_1_ and *PignisticVector m*_2_ , and |*PignisticVector m*| refers to the length of the vector.

Because the Pignistic probability should be limited to interval [0, 1], the angle between PignisticVectors will be [0°, 90°], and the corresponding cosine values should be [0, 1]. When the angle is 0°, the cosine value is 1, which infers that the two pieces of evidence are fully supported. When the angle is 90°, the cosine value is 0, which means the degree of conflict is the largest. As the cosine function is monotonically decreasing in the domain [0, $\overset{\pi }{\;}/\underset{2}{\;}$], the cosine value decreases with an increase in the angle between vectors.

To express the degree of conflict between pieces of evidence, this article uses the following definition:

(11) $$\text{ConfDegree}\left(m_{1},m_{2}\right)=1-\cos\left(m_{1},m_{2}\right)$$

*ConfDegree* denotes the degree of conflict between evidence *m*_1_ and *m*_2_, and the conflicting value *ConfDegree* monotonically increases with an increase of the degree of conflict. When the belief assignment results for two pieces of evidence are the same, we can obtain *ConfDegree* = 0, whereas, when the pieces of evidence conflict completely *ConfDegree* = 1.

Specifically, the *n*-dimensional pignistic probability space vector converted by Formula (9) may impact the real reliability allocation results.

### Example 2

Under the frame of discernment of Θ = {*A*, *B*}, the basic reliability allocation results of evidence *E*1, *E*2 are shown as follows:

$$\begin{array}{cccc} E1: & m_{1}\left(A\right)=0.5, & m_{1}\left(B\right)=0.5, & m_{1}\left(\Theta \right)=0\\ E2: & m_{2}\left(A\right)=0, & m_{2}\left(B\right)=0, & m_{2}\left(\Theta \right)=1 \end{array}$$

Using Equation (9), we can get *PignisticVe*c*tor m*_1_ = [0.5, 0.5]*^T^*, *PignisticVe*c*tor m*_2_ = [0.5, 0.5]*^T^*. We find that cos(*m*_1_, *m*_2_) = 1 through Equation (9), even though the initial reliability allocation results of evidence *E*1 and *E*2 are different.

The above result seems like violating the analysis principle of the conflict data. But, due to *m*_2_ (Θ) = 1, it means *E*2 does not know the concrete reliability allocation results. According to the uncertainty principle of retaining unknown information in maximum entropy theory, the result of *m*_2_ (*A*) = 0.5, *m*_2_ (*B*) = 0.5 is reasonable.

Inevitably, the existence of mixed set also can lead to situation that the *PignisticVe*c*tors* are the same. Under these conditions, we could calculate the cosine of the initial reliability allocate results and without the mixed set. Then, comparing the value of cosine with the cos(*PignisticVe*c*tor*). If these two values are pretty inconsistent, we consider that the procedure of reducing the dimension is not necessary. The cosine could be calculated just by the initial reliability allocation results.

## 4. Evidence Fusion Algorithm Based on Degree of Support between Pieces of Evidence

In Section 3, the method for computing the degree of support between pieces of evidence was defined. To make further efforts, an evidence fusion algorithm is proposed based on the degree of support, which is calculated using the cosine of the pieces of evidence. The constructed correlation matrix considers the relativity among pieces of evidence. As a result, the proposed algorithm has a stronger ability to eliminate conflict between the pieces of evidence.

According to the obtained masses, the presented algorithm calculates the angle between the pieces of evidence, which is the metric for the degree of support between them. Then, we calculate the mean value of the weighted credibility by using Deng’s method. Finally, we apply Murphy’s method to fuse the data and obtain the fused masses. On basis of the results, we can consider whether it is necessary to add new evidence or perfect the frame for the discernment.

Using Expression (10), the angle between two pieces of evidence can be obtained. Assume that there are *k* data sources. After calculating the degrees of support, the correlation matrix can be constructed as follows:

(12) $$\left[S=\left[\begin{array}{cccc} 1 & \cos\left(m_{1},m_{2}\right) & \cdots & \cos\left(m_{1},m_{k}\right)\\ \cos\left(m_{2},m_{1}\right) & 1 & \cdots & \cos\left(m_{2},m_{k}\right)\\ \cdots & \cdots & \cdots & \cdots \\ \cos\left(m_{k},m_{1}\right) & \cos\left(m_{k},m_{2}\right) & \cdots & 1 \end{array}\right]\right]$$

In the correlation matrix *S*, the elements on the diagonal are the degrees of support, which are all equal to 1. The sum of all the elements in row *i* is the degree of support for evidence *i*. The support vector for *k* pieces of evidence is shown in the following expression:

(13) $$\left[\text{Sup}=\left(\begin{array}{c} \overset{k}{\underset{j=1}{\text{∑}}}\cos\left(m_{1},m_{j}\right)\\ \overset{k}{\underset{j=1}{\text{∑}}}\cos\left(m_{2},m_{j}\right)\\ \vdots \\ \overset{k}{\underset{j=1}{\text{∑}}}\cos\left(m_{i},m_{j}\right)\\ \vdots \\ \overset{k}{\underset{j=1}{\text{∑}}}\cos\left(m_{k},m_{j}\right) \end{array}\right)\right]$$

In *Sup*, *Sup* (i) indicates the degree of support for evidence *i*, where a larger value indicates greater similarity. To obtain reasonable weights for the pieces of evidence, Expression (13) should be normalized as follows:

(14) $$\left[\text{Sum}\left(\text{Sup}\right)=\overset{k}{\underset{i=1}{\text{∑}}}\text{Sup}\left(i\right)\right]$$

(15) $$\left[\text{Crd}=\frac{1}{\text{Sum}\left(\text{Sup}\right)}\times \text{Sup}\right]$$

Obviously, there is $\overset{k}{\underset{i=1}{\text{∑}}}\text{Crd}\left(i\right)=1$, where *Crd*(*i*) can be used as the weight of evidence *m_i_*, and the average credibility can be obtained as:

(16) $$\left[\text{MAE}\left(m\right)=\overset{k}{\underset{i=1}{\text{∑}}}\text{Crd}\left(i\right)\times m_{i}\right]$$

It’s important to note that the proposed algorithm adds up all the elements in a row, including the elements on the diagonal (all equal to 1). This is different from Deng’s method, which does not include the diagonal elements. Intuitively, the result calculated by our method can better reflect the real support degree of the mutual evidence.

For example: suppose that there are three evidences *m*_1_, *m*_2_ and *m*_3_, and the correlation matrix S:

$$\text{S}=\left(\begin{array}{ccc} 1 & 0 & 0\\ 0 & 1 & 0.1\\ 0 & 0.1 & 1 \end{array}\right)$$

In this example, the evidence *m*_1_ is completely conflict with *m*_2_ and *m*_3_, and the degree of evidence conflict between *m*_2_ and *m*_3_ is also great. With the method proposed by Deng *et al.*, the weight of *m*_1_ is 0, and the weights of *m*_2_ and *m*_3_ both are 0.5. This is unreasonable.

The main steps of the algorithm are shown below.

(1) Calculate the correlation coefficients and construct correlation matrix S. After the Pignistic transfer process, the number of credibility assignments will decrease from 2^n^-dimension to the n-dimension. With the use of the cosine theorem, the degrees of support for the pieces of evidence will be obtained, after which correlation matrix S is constructed.
(2) Calculate the support vector and credibility assignments for the pieces of evidence. By using Expressions (12)–(14), the support vectors and credibility values for the pieces of evidence will be obtained. In a situation with a high degree of conflict for one piece of evidence, it is unreasonable to only use other pieces of evidence to calculate its degree of support. Therefore, it is included in Expression (12) to avoid the occurrence of very low support.
(3) Calculate the weighted mean of the credibility assignments by using the obtained degrees of support and credibility values. The transferred credibility assignments can be calculated using Expression (15).
(4) Fuse the obtained credibility by using the Dempster’s combination rule and Murphy’s method. In Murphy’s method, the evidence’s mass is just reformed by averaging. Here, we reform the mass using a weighted mean, which not only benefits from the advances of Murphy’s method in dealing with conflicting pieces of evidence with a high calculation speed, but also takes into account the correlation between pieces of evidence.
(5) Under the Open World and Close World Assumption, make the decision based on the data fusion results.

As we all know, the high conflict of the data is equivalent to the great difference of the reliability allocation on the same assumption. Because we adopt the evidence weighted average fusion method in this paper, there is maybe a potential problem. If the difference of the reliability allocation, which is obtained after the fusion operation of the high conflict data is less than the preset difference threshold *th*, it is difficult to make the decision. In particular, the value of *th* is set based on the actual situation. In order to make the decision, under the Close World Assumption, we need increase the number of evidences. And under the Open World Assumption, we need to add new assumptions in the frame of discernment.

**Figure 2.**
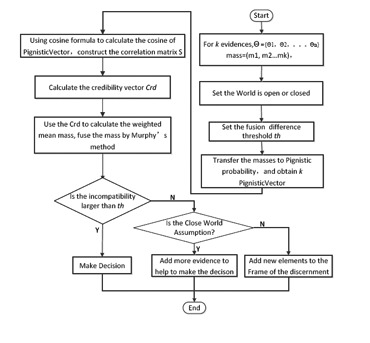
Flowchart of proposed method.

The flowchart of the process is given in Figure 2.

**Pseudo random code is shown below:**

**Input:** 1.Mass (***m*_1_**, ***m*_2_**, ***m*_3_**,…***m*_k_**), reliability allocation results of k pieces of evidence in the sample space Θ = {Θ_1_, Θ_2_,…, Θ_3_}; 2.the condition of the open world assumption ***isCWA***, if ***isCWA* = 1** means Close World Assumption, otherwise means Open World Assumption; 3.***th***, the threshold of the fusion result difference.**Output:** the result of decision judgement**Procedure:** 1:**For each**
*i* ∈ *k* // *k* is the number of evidence 2:
$\text{Pignistvector}\;i={\text{∑}}_{A\subseteq \Theta ,A\cap \Theta j\neq \phi }\frac{1}{\left|A\right|}\frac{m\left(A\right)}{1-m\left(\phi \right)}$ // Pignistvector *i* is the Pignistic probability vector of *i*, transited from a 2*^n^*-dimensional vector to an n-dimensional vector 3:**End for** 4:**For each**
*i* ∈ *k* , *j* ∈ *k* 5:
$\cos\left(i,j\right)=\frac{<\text{PignisticVector}\;i,\;\text{PignisticVector}\;j>}{\left|\text{PignisticVector}\;i\right|\times \left|\text{PignisticVector}\;j\right|}$ // get the angle of two PignisticVector 6:**End for** 7:
$\text{Sup}\left[i\right]={\text{∑}}_{j=1}^{k}\cos\left(i,j\right)$ // *Sup*[*i*] indicates the degree of support for evidence *i* 8:
$\text{SumSup}={\text{∑}}_{j=1}^{k}\text{Sup}\left[i\right]$ // *SumSup* is the degree of support for all the evidence, prepared for normalization 9:
$\text{Crd}\left[i\right]=\frac{1}{\text{SumSup}}\text{Sup}\left[i\right]$ // normalized 10:
$\text{MAE}\left(m\right)={\text{∑}}_{i=1}^{k}Crd\left(i\right)*m_{i}$ // MAE(*m*) is the weighted average credibility of the original reliability 11:*t* = Murphy(MAE) // using Murphy’s method to average the credibility 12:**If** (*t* > *th*) // *t* is the incompatibility computed above 13:**Then** 14:Make decision 15:**Else if** (*isCWA* = 1) 16:**Then** 17:add more evidence to make the decision 18:**Else** 19:add new elements to the frame of the discernment 20:**end procedure**


## 5. Experiments Results

### 5.1. Results for Degree of Conflict Based on Cosine Theorem

The proposed method defined a new way to measure the conflict between pieces of evidence, and also the degree of support. It is essential to the method, so it is necessary to validate whether it is reasonable to show the conflict level.

**Figure 3.**
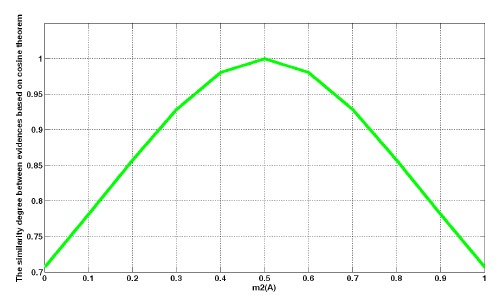
The similarity of the evidences *E*1 and *E*2 when *m*_1_(*A*)=0.5, *m*_1_(*B*) = 0.5.

Given the discernment frame Θ = {*A, B*}, there are two pieces of evidence with the credibility assignments shown below:

$$\begin{array}{cccc} E1: & m_{1}\left(A\right)=0.5, & m_{1}\left(B\right)=0.5, & m_{1}\left(\Theta \right)=0\\ E2: & m_{2}\left(A\right)=x, & m_{2}\left(B\right)=1-x, & m_{2}\left(\Theta \right)=0 \end{array}$$

where *x* denotes the credibility of hypothesis *A*, and the credibility of hypothesis *B* is 1−*x*. With an increase in *x*, the degree of support between the two pieces of evidence is as shown in Figure 3. The *x*-axis is the results of the reliability allocation. The *y*-axis is the similarity of the evidences *E*1 and *E*2.

As shown in Figure 3, when *x* increases from 0 to 0.5, the degree of support increases from 0.7071 to 1.0. In contrast, in the interval [0.5, 1], the degree of support decreases with an increase in *x*, and the cosine curve line is symmetrical with *x* = 0.5. The change is reasonable to show the relativity between pieces of evidence.

When the conflict level is large, to simulate this situation, we assume that the credibility values of *E*1 and *E*2 are as follows:

$$\begin{array}{cccc} E1: & m_{1}\left(A\right)=1, & m_{1}\left(B\right)=0, & m_{1}\left(\Theta \right)=0\\ E2: & m_{2}\left(A\right)=x, & m_{2}\left(B\right)=1-x, & m_{2}\left(\Theta \right)=0 \end{array}$$

**Figure 4.**
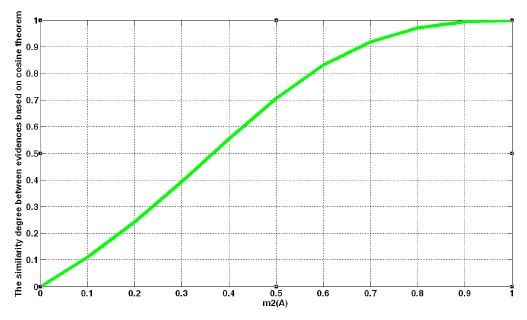
Curve line for degree of support when *x* increases from 0 to 1.

The obtained degree of support is shown in Figure 4.

Figure 4 illustrates the variation in the degree of support with different values of *x*. The cosine curve increases from 0 to 1 when *x* increases from 0 to 1. The variation trend is reasonable and meaningful to show the degree of support.

**Figure 5.**
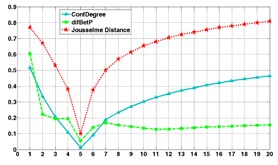
The curve lines of the conflict based on the results of the three methods.

Next, we discuss a comparison simulation of the three methods for measuring conflicts: *d_BPA_* based on the Jousselme Distance, *difBetP* based on Pignistic probability, and *ConfDegree* based on the proposed method. In [2], the situation is given as Θ = {Θ_1_, Θ_2_,…, Θ_20_}, and the credibility assignments for pieces of evidence *E*1 and *E*2 are as follows:

$$\begin{array}{l} \begin{array}{ll} E1: & \begin{array}{cccc} m_{1}\left(\Theta_{2},\Theta_{3},\Theta_{4}\right)=0.05, & m_{1}\left(\Theta_{7}\right)=0.05, & m_{1}\left(\Theta \right)=0.1, & m_{1}\left(A\right)=0.8 \end{array}\\ E2: & m_{2}\left(1,2,3,4,5\right)=1 \end{array}\\ \begin{array}{ll} E1: & \begin{array}{cccc} m_{1}\left(\Theta_{2},\Theta_{3},\Theta_{4}\right)=0.05, & m_{1}\left(\Theta_{7}\right)=0.05, & m_{1}\left(\Theta \right)=0.1, & m_{1}\left(A\right)=0.8 \end{array}\\ E2: & m_{2}\left(\Theta_{1},\Theta_{2},\Theta_{3},\Theta_{4},\Theta_{5}\right)=1 \end{array} \end{array}$$

where *A* is contained in Θ, and Θ is a changeable set. First, we set *A* = {Θ_1_}, then, adding a new element to subset *A* each step until *A* = {Θ_1_, Θ_2_,…, Θ_20_}. The change process of set *A* has shown in Table 1:

With the increase in the elements in *A*, the conflict between two pieces of evidence is shown in Figure 5.

With an increase in the elements in subset *A*, the conflict level changes according to the different *A*. It is apparent in Step 1 to Step 5 that the credibility assignments of *E*1 in set {Θ_1_, Θ_2_, Θ_3_, Θ_4_, Θ_5_} gradually increase, and the degree of conflict decreases. Then, after Step 5, as the elements increase, the credibility assignments of *E*1 in set {Θ_1_, Θ_2_, Θ_3_, Θ_4_, Θ_5_} gradually decrease, and the conflict increases. These results prove that this is also an effective and reasonable way to measure the conflict between pieces of evidence.

Compared to the two existing methods, the proposed method is similar in the change trend of Jousselme’s method, and the two methods are more reasonable for showing the conflicts. Moreover, the amount of calculation needed for the proposed method is lower than that for Jousselme’s method. The method based on the Pignistic probability has a very low computational complexity. However, it does not show the increasing trend after Step 8, which shows that it is not sensitive to the change in the conflict between pieces of evidence.

### 5.2. Fusion Results Based on Degree of Support between Pieces of Evidence

To verify the rationality and validity of the proposed fusion method based on the degree of support, we use the example in [14] to test the algorithm and analyze the results.

Assume that there are three objects in a target recognition system. Thus, the discernment frame is Θ = {*A*, *B*, *C*}. There are five different kinds of sensors to observe the object. They are a CCD sensor (*S*1), sound sensor (*S*2), infrared sensor (*S*3), radar (*S*4), and ESM sensor (*S*5). Now, there is an object that belongs to *A*, and the pieces of evidence obtained from the five kinds of sensors are as follows:

$$\begin{array}{ll} S1: & \begin{array}{ccc} m_{1}\left(A\right)=0.41, & m_{1}\left(B\right)=0.29, & m_{1}\left(C\right)=0.3 \end{array}\\ S2: & \begin{array}{ccc} m_{2}\left(A\right)=0, & m_{2}\left(B\right)=0.9, & m_{2}\left(C\right)=0.1 \end{array}\\ S3: & \begin{array}{ccc} m_{3}\left(A\right)=0.58, & m_{3}\left(B\right)=0.07, & m_{3}\left(AC\right)=0.35 \end{array}\\ S4: & \begin{array}{ccc} m_{4}\left(A\right)=0.55, & m_{4}\left(B\right)=0.1, & m_{4}\left(AC\right)=0.35 \end{array}\\ S5: & \begin{array}{ccc} m_{5}\left(A\right)=0.6, & m_{5}\left(B\right)=0.1, & m_{5}\left(AC\right)=0.3 \end{array} \end{array}$$

In this situation, the perception of the sound sensor (*S*2) is abnormal, which may cause wrong fusion results. Table 2 lists the fusions results when using different combination rules.

In Table 2, the fusion results with the Dempster combination rule are abnormal when *S*2’s evidence conflicts with the other sensors, which causes the wrong decision results. This shows that the Dempster combination rule will produce counter-intuitive results, and the abnormal evidence accounts for a veto to the fusion results. When more pieces of evidence are included, the combination rules of Yager, Murphy, Deng, and the proposed method all produce accurate and reasonable results, which solves the “veto problem” effectively. Yager’s method has a lower convergence speed than the other two methods. Murphy’s method just reforms the pieces of evidence using simple averaging, and it is sensitive to a change in a single piece of evidence. Deng’s method and the proposed method both take the approach of weighted averaging. Their change trends and convergence speeds are similar. However, the proposed method is easier to use and requires a lower amount of calculation.

To validate the rationality of the proposed method, we perform experiments in different situations. Group 1 includes two pieces of evidence with a high degree of conflict. Group 2 includes two pieces of evidence with low conflict, and Group 3 includes three pieces of evidences with high degrees of conflict. The discernment frame is Θ = {*A*, *B*, *C*}. In Table 3, the fusion results for Groups 1 and 3 show that the proposed algorithm is able to effectively avoid the Zadeh paradox. With different pieces of evidence, the fusion results will get a more reasonable credibility assignment. The proposed method’s results are also reasonable in a low-conflict situation. The experimental results prove that the proposed method is a rational and effective way to detect and solve the conflict between pieces of evidence in data fusion by using the DS evidence theory.

## 6. Conclusions

In this paper, the main problems in data fusion were first briefly introduced. Considering the deep influence of conflicts between pieces of evidence on the fusion results, a new method based on the cosine theorem was developed to measure the conflicts between pieces of evidence. The proposed method is easy to implement and is able to show the degree of support between pieces of evidence. Moreover, this paper presented a new improved evidence fusion algorithm based on the degree of support. Experimental results proved that the proposed method is able to produce reasonable fusion results. It is an effective way to deal with the Zadeh paradox, veto problem, and fair credibility assignment problem.

At the same time, there are still problems that need to be solved. In this paper, the index explosion in the DS evidence theory was not taken into consideration. On the other hand, in the weighted mean step using the degree of support, we just considered the degree of support between the different pieces of evidence and ignored the influence of other factors. In a future study, the improvements may include the following: (1) applying the degree of support to develop a flexible and effective new way to deal with the index explosion problem and (2) improving the weighted mean step and considering additional influence factors.

## Figures and Tables

**Table 1.** Change process of set *A* in each step.

**Step**	***A***
1	{Θ_1_}
2	{Θ_1_, Θ_2_}
3	{Θ_1_, Θ_2_, Θ_3_}
4	{Θ_1_, Θ_2_, Θ_3_, Θ_4_}
5	{Θ_1_, Θ_2_, Θ_3_, Θ_4_, Θ_5_}
⋮	⋮
20	{ Θ_1_, Θ_2_, Θ_3_, ⋯ , Θ_20_}

**Table 2.** Fusion results with different combination rules.

**Combination Rule**	**Fusion Results**			

	*m*_1_, *m*_2_	*m*_1_, *m*_2_, *m*_3_	*m*_1_, *m*_2_, *m*_3_, *m*_4_	*m*_1_, *m*_2_, *m*_3_, *m*_4_, *m*_5_
Dempster	*m*(*A*) = 0	*m*(*A*) = 0	*m*(*A*) = 0	*m*(*A*) = 0
	*m*(*B*) = 0.8969	*m*(*B*) = 0.6575	*m*(*B*) = 0.3321	*m*(*B*) = 0.1422
	*m*(*C*) = 0.1031	*m*(*C*) = 0.3425	*m*(*C*) = 0.6679	*m*(*C*) = 0.8578

Yager	*m*(*A*) = 0	*m*(*A*) = 0.4112	*m*(*A*) = 0.6508	*m*(*A*) = 0.77323
	*m*(*B*) = 0.261	*m*(*B*) = 0.0679	*m*(*B*) = 0.033013	*m*(*B*) = 0.01669
	*m*(*C*) = 0.03	*m*(*C*) = 0.0105	*m*(*C*) = 0.00367	*m*(*C*) = 0.00110
		*m*(*AC*) = 0.2481	*m*(*AC*) = 0.178633	*m*(*AC*) = 0.09375
	*m*(*θ*) = 0.709	*m*(*θ*) = 0.2622	*m*(*θ*) = 0.133872	*m*(*θ*) = 0.11523

Murphy	*m*(*A*) = 0.0964	*m*(*A*) = 0.4619	*m*(*A*) = 0.8362	*m*(*A*) = 0.9620
	*m*(*B*) = 0.8119	*m*(*B*) = 0.4497	*m*(*B*) = 0.1147	*m*(*B*) = 0.0210
	*m*(*C*) = 0.0917	*m*(*C*) = 0.0794	*m*(*C*) = 0.0410	*m*(*C*) = 0.0138
	*m*(*AC*) = 0	*m*(*AC*) = 0.0090	*m*(*AC*) = 0.0081	*m*(*AC*) = 0.0032

Deng *et.al.*	*m*(*A*) = 0.0964	*m*(*A*) = 0.4974	*m*(*A*) = 0.9089	*m*(*A*) = 0.9820
	*m*(*B*) = 0.8119	*m*(*B*) = 0.4054	*m*(*B*) = 0.0444	*m*(*B*) = 0.0039
	*m*(*C*) = 0.0917	*m*(*C*) = 0.0888	*m*(*C*) = 0.0379	*m*(*C*) = 0.0107
	*m*(*AC*) = 0	*m*(*AC*) = 0.0084	*m*(*AC*) = 0.0089	*m*(*AC*) = 0.0034

Proposed method	*m*(*A*) = 0.0964	*m*(*A*) = 0.568114	*m*(*A*) = 0.91420	*m*(*A*) = 0.98199
	*m*(*B*) = 0.8119	*m*(*B*) = 0.331892	*m*(*B*) = 0.039475	*m*(*B*) = 0.00339
	*m*(*C*) = 0.0917	*m*(*C*) = 0.092942	*m*(*C*) = 0.039858	*m*(*C*) = 0.01145
	*m*(*AC*) = 0	*m*(*AC*) = 0.00844	*m*(*AC*) = 0.00826	*m*(*AC*) = 0.00317

**Table 3.** Fusion results for proposed method in different situations.

	**Group 1**	**Group 2**	**Group 3**
Pieces of evidence			*m*_1_(*A*) = 0.99
	*m*_1_(*A*) = 0.99	*m*_1_({*AB*}) = 0.5	*m*_1_(*B*) = 0.01
	*m*_1_(*B*) = 0.01	*m*_1_(*C*) = 0.5	*m*_2_(*B*) = 0.01
	*m*_2_(*B*) = 0.01	*m*_2_(*A*) = 0.5	*m*_2_(*C*) = 0.99
	*m*_2_(*C*) = 0.99	*m*_2_({*BC*}) = 0.5	*m*_3_(*B*) = 0.99
			*m*_3_(*C*) = 0.01
Fusion results		*m*(*A*) = 0.3	
	*m*(*A*) = 0.4999	*m*(*B*) = 0.2	*m*(*A*) = 0.3130191
	*m*(*B*) = 0.0002	*m*(*AB*) = 0.1	*m*(*B*) = 0. 3524118
	*m*(*C*) = 0.4999	*m*(*C*) = 0.3	*m*(*C*) = 0.3324744
		*m*(*BC*) = 0.1	
